# A case report about eosinophilic enteritis presenting as abdominal pain

**DOI:** 10.1097/MD.0000000000027296

**Published:** 2021-10-01

**Authors:** Hairong Zhang, Cuimei Ma, Yuan Xue, Zongjing Hu, Zhen Xu, Yibo Wang, Guangxi Zhou

**Affiliations:** a Department of Gastroenterology, Affiliated Hospital of Jining Medical University, Jining Medical University, Jining, Shandong, P.R. China; b Institute of Digestive Endoscopy, Jining Medical University, Jining, Shandong, P.R. China.

**Keywords:** diagnosis, eosinophilic enteritis

## Abstract

**Rationale:**

Eosinophilic enteritis (EE) is an immune-mediated antigen-driven disease that may lead to clinical symptoms and organ dysfunction and characterized by the presence of extensive eosinophilic infiltrates on histopathological examination of the intestinal mucosa.

**Patient concerns:**

A 29-year-old man presented with a half-month duration of paroxysmal upper abdominal pain that gradually evolved into continuous pain accompanied by the urge to defecate.

**Diagnoses:**

Pathological findings of enteroscopy showed acute and chronic inflammation accompanied by eosinophilic infiltration (>20/ high-power field).

**Interventions:**

The patient was initially treated with IV infusion of dexamethasone 10 mg per day for 3 days, which was reduced to 7.5 mg per day for 2 days once pain relief was achieved. Upon discharged from our hospital, the patient was prescribed with oral prednisolone 30 mg per day, which was reduced by 5 mg per week for 6 weeks until discontinuation.

**Outcomes:**

The patient was relieved from the pain after receiving dexamethasone for 5 days, and he was maintained on oral prednisolone 30 mg per day upon discharge from the hospital. On the day of discharge, the eosinophil count and derived ratios were normal.

**Lessons:**

In patients with EE, the dynamic changes of the eosinophil count should be monitored. Clinicians must be aware that not all patients with EE have a history of allergies. In the management and treatment of the disease, multisite biopsies should be carried out if EE is suspected, and EE is responsive to steroid therapy.

## 1 Introduction

Eosinophilic gastrointestinal disorders are rare conditions characterized by excess eosinophils in the mucosal biopsies of 1 or multiple sites in the gastrointestinal tract in the absence of other known causes of tissue eosinophilia that may lead to organ dysfunction and clinical symptoms.^[1,2]^ These conditions include eosinophilic esophagitis, eosinophilic gastritis, eosinophilic gastroenteritis, eosinophilic enteritis (EE), and eosinophilic colitis.^[3]^ EE, a more common form of eosinophilic gastrointestinal disorders, is characterized histopathologically by the presence of extensive eosinophilic infiltrates in the intestinal mucosa histopathologically.^[4,5]^ Despite several epidemiological and clinical features suggesting an allergic component, its etiology is still unknown.^[6]^ EE causes an array of gastrointestinal symptoms such as abdominal pain, diarrhea, nausea, vomiting, abdominal bloating, or ascites. The diagnosis of EE is based upon a high degree of clinical likelihood, nonspecific clinical presentation, physical examination findings, and demonstration of pathological eosinophilic infiltration.^[7]^ Here, we report a case of EE presenting as abdominal pain, which has not been reported in published studies and provide a review of related literatures. This study was approved by the Institutional Review Board for Clinical Research of the Affiliated Hospital of Jining Medical University. Written informed consent was also obtained from the patient before study.

## 2 Case presentation

**Figure 1:**
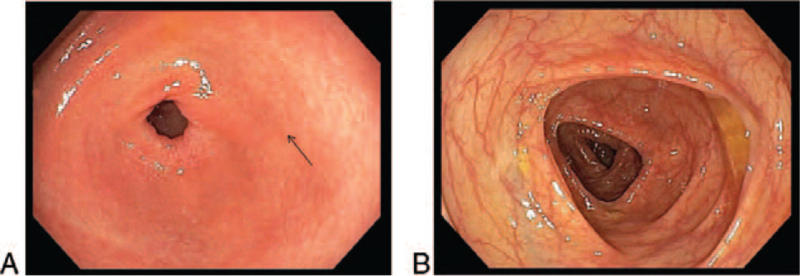
Gastroscopy (A) and colonoscopy (B) images. Gastroscopy revealed gastritis, whereas colonoscopy showed a normal mucosa.

A 29-year-old man presented with a half-month history of paroxysmal upper abdominal pain that gradually evolved into continuous pain and was accompanied by the urge to defecate. He went to the local hospital on June 9 where he underwent the following diagnostic investigations: upper abdominal computerized tomography (CT) scan, routine blood tests, gastroscopy, and colonoscopy. No abnormalities were observed on CT. The results of routine blood tests are shown in Table 1. Gastroscopy revealed gastritis, whereas colonoscopy showed a normal colonic mucosa (Fig. 1). After symptomatic treatment, the patient experienced slight relief from the pain.

**Table 1:** The results of blood routine examination.

Date	WBC (×10^9^/L)	N (%)	EO (%)	EO (×10^9^/L)	RBC (×10^12^/L)	HGB (g/L)	PLT (×10^9^/L)
06.09.2020	7.2	63	0.82	0.56	4.71	146	256
06.18.2020	10.7	61.5	9.1	0.98	5.67	178	198
06.21.2020	9.96	59.2	12.2	1.22	5.45	170	199
06.28.2020	13.99	73.9	0.8	0.11	4.91	152	243
07.06.2020	14.19	61.6	3.2	0.64	5.21	170	224
10.12.2020	8.40	58.40	1.80	0.15	3.83	128	252

EO = eosinophil, HGB = hemoglobin, N = neutrophils, PLT = platelet, RBC = red blood cell, WBC = white blood cell.

On June 16, the pain worsened; it was persistent and accompanied by the urge to defecate. He defecated once a day, and his stools were characterized as loose and non-bloody. He often experienced abdominal distention and heartburn, and he had not lost any weight recently. For further evaluation and treatment, he was admitted into our hospital (the affiliated hospital of Jining Medical College) on June 18, 2020. The patient's medical history did not reveal any past diseases, and he had no history of drug abuse or food allergy. The physical examination was unremarkable.

The results of the routine blood tests performed on June 18 are shown in Table 1. On June 19, the appendiceal ultrasound showed normal results, whereas the chest CT scan findings indicated bronchitis. Magnetic resonance cholangiopancreatography did not reveal cholangiectasis or cholecystitis. The results of the liver function test, kidney function test, serum calcium, serum electrolyte test, blood glucose, urine routine test, and antinuclear antibody spectrum were all normal. In addition, no parasitic ovum was found in the stool ova and parasites test. However, the fecal occult blood test was positive.

The initial diagnosis that was considered was gastritis and a disorder of the gastrointestinal function. Hence, we administered omeprazole, otilonium bromide, and probiotics along with other symptomatic and supportive treatments. However, the abdominal pain gradually worsened to the point where spasmolytics and pain reliever (tramadol hydrochloride and 654-2) were not effective anymore.

**Figure 2:**
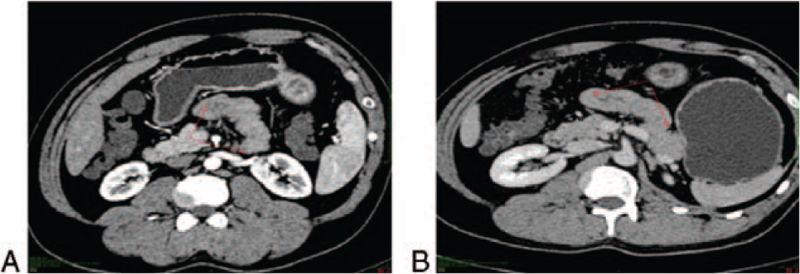
Enhanced CT images. The images show diffuse thickening of the jejunum wall (arrows), which was suspected to be inflammation, although the presence of a tumor could not be ruled out completely. CT = computerized tomography.

**Figure 3:**
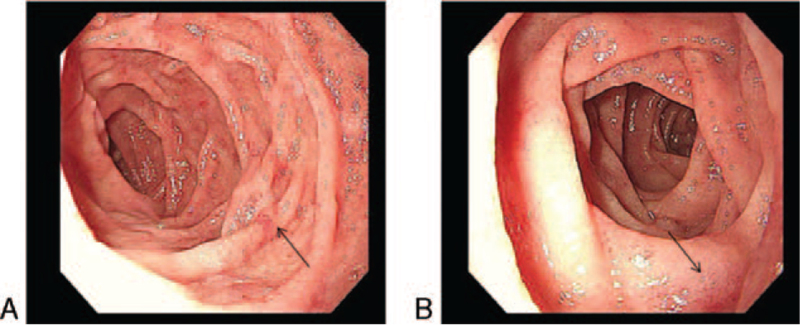
The enteroscopy images. The duodenal mucosa was congested and eroded especially in the descending and horizontal part of the duodenum (arrows).

**Figure 4:**
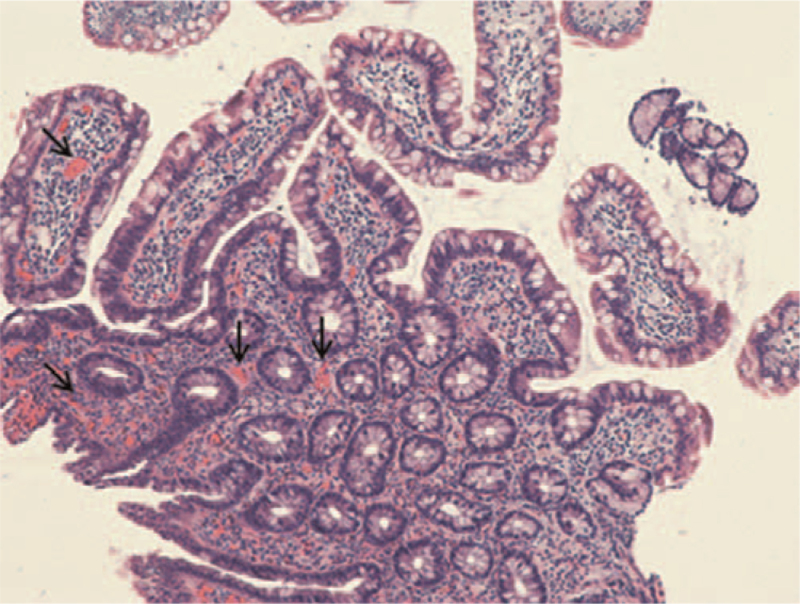
Pathological findings in the lamina propria. Pathological findings show acute and chronic inflammation accompanied by eosinophilic infiltration (>20/HPF) (arrows). HPF = high-power field.

**Figure 5:**
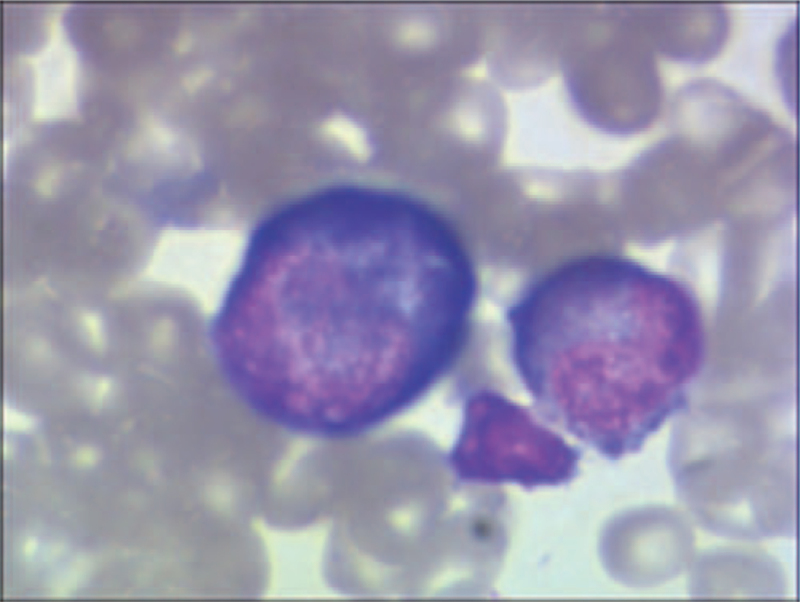
The bone marrow smear findings. The ratio of eosinophils was slightly higher, but the cells were morphologically normal.

**Figure 6:**
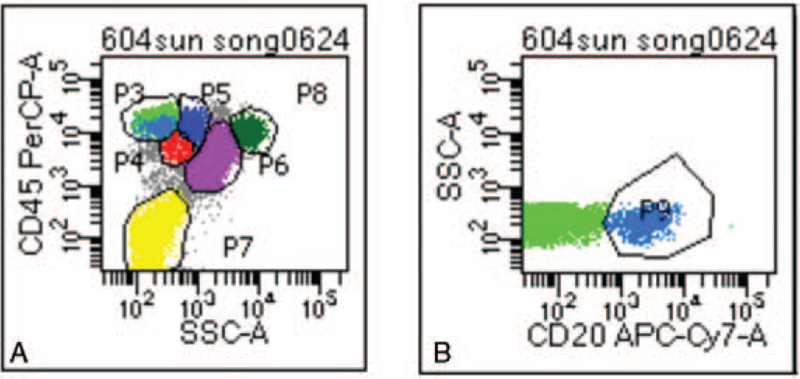
Classification and phenotypes of nucleated cells and lymphocytes by flow cytometry. There was no abnormal lymphocyte phenotype. SSC-A = side scatter area.

On June 21, the enhanced CT scan showed diffuse thickening of the jejunum wall which was suspected to be due to inflammation, although the presence of a tumor could not be ruled out completely (Fig. 2). The blood routine tests in Table 1 were repeated, and the results revealed an eosinophil count of 1.22 × 10^9^/L, which was higher than the previous results. In addition, the ratio of eosinophils was 12.2% higher than that of the previous results. Thus, we arrived at a diagnosis of EE. On June 22, an enteroscopy was performed, which showed gastritis, congestion of the duodenal and jejunal mucosa, and partial erosion of the mucosal membrane, especially in the descending part of the duodenum (Fig. 3). Biopsy specimens were obtained from the descending duodenum, horizontal segment of the duodenum, and jejunum. In all biopsy specimens, the pathological findings showed acute and chronic inflammation accompanied by eosinophilic infiltration (>20 eosinophils/ high-power field [HPF]) (Fig. 4). On June 23, bone marrow examination was performed, and the results revealed a slightly higher ratio of eosinophils with a preserved cellular morphology (Fig. 5). The subsequent flow cytometry did not reveal abnormal lymphocyte phenotypes (Fig. 6, Tables 2 and 3).

**Table 2:** Classification of nucleated cells.

Classification of nucleated cells	% of nuclear cells
Lymphocyte (P3)	7.68%
Monocyte (P5)	3.44%
Granulocyte (P6)	62.45%
Eosinophils (P9)	7.49%
Nucleated red blood cell (P8)	14.60%
Medullary immature cells (P4)	1.45%

**Table 3:** Classification of lymphocyte.

Classification of lymphocyte	% of lymphocyte
B cell (CD19^+^)	14.13%
T cell (CD3^+^)	59.85%
NK lymphocyte (CD5^−^ CD7^+^)	24.73%

NK = Natural killer.

Based on the clinical manifestations, CT scan and enteroscopy images, pathological findings, and the results of bone marrow examination and flow cytometry, we considered EE as the most likely diagnosis. On June 24, we initiated dexamethasone 10 mg IV infusion per day for 3 consecutive days; the abdominal pain was resolved on the first day. Three days later, we reduced the dose of dexamethasone to 7.5 mg per day for 2 days. After 5 days since initiating dexamethasone, the patient was discharged from our hospital, and oral prednisolone (30 mg per day) was prescribed. On the day of discharge, we repeated the routine blood test and found that the eosinophil count and EO ratio derived ratios returned to the normal ranges (0.11 × 10^9^/L and 0.8%, respectively) (Table 1). But before the steroid giving to the patient, eosinophil counts were 1.22 × 10^9^/L, and EO ratio was 12.2%, they were both higher than normal (Table 1). Since our patient had no history of food allergy, we did not modify the patient's diet.

After the patient was discharged, we informed him to taper the dose of prednisolone by 5 mg per week. On July 6, he returned to our hospital for a follow-up. He was asymptomatic and underwent routine blood tests, with the results shown in Table 1. The eosinophil count and EO ratio were 0.64 × 10^9^/L and 3.2%, respectively, which were both normal. During the follow-up, he had been taking prednisolone 20 mg per day. During the following month, the dosages of steroids were gradually tapered until discontinuation. The total dosage taken for each steroid was 45 mg and 735 mg for dexamethasone and prednisolone, respectively. On October 12, a telephone follow-up revealed that he had completely stopped taking steroids and had no obvious symptoms. The results of the repeat routine blood tests are shown in Table 1. The eosinophil counts and EO ratio were 0.15 × 10^9^/L and 1.8%, respectively.

## 3 Discussion

EE is an immune-mediated antigen-driven disease characterized by the presence of extensive eosinophilic infiltrates on histopathological examination of the intestinal mucosa that may manifest clinical symptoms and cause organ dysfunction.^[4,8]^ Although it can manifest at any age, EE typically presents in the third to the fifth decade and has a peak age of onset in the third decade, with patients being predominantly male.^[9–11]^ EE is a rare condition that was first described by Kaiser in 1937, and its prevalence in the general population is estimated to be 1 per 100,000. Until recently, less than 400 cases have been reported in the literature.^[8]^ The most common symptoms of EE include abdominal pain, nausea, vomiting, dysphagia, diarrhea, early satiety, and weight loss; however, serious manifestations such as intestinal obstruction or perforation may occur.^[3]^

The diagnosis of EE is based on the nonspecific clinical presentation and results of auxiliary examinations. The peripheral eosinophil count is usually elevated and ranges from 5% to 35%.^[9]^ It has been reported that 80% of patients with EE had elevated peripheral eosinophil counts.^[9,12]^ Endoscopy is essential in the diagnosis of EE, but the endoscopic findings such as mucosal erythema, friability, nodularity, polyps, edema, ulceration, and fibrosis, as well as the complete loss of villi are nonspecific.^[9,13,14]^ In our case, we only observed erythema and mucosal erosion. The pathological diagnostic criteria dictates that there should be more than 15 to 50 eosinophils per HPF in the lamina propria on histological examination from at least 6 different biopsy sites.^[15]^ In this patient, the results of the routine blood test at the peak of disease showed that the ratio of eosinophils was markedly increased, and the pathological examination demonstrated eosinophilic infiltration of more than 20 eosinophils/HPF in the lamina propria. Moreover, the elevated ratio of eosinophils was also seen in the bone marrow. These results supported the diagnosis of EE in this patient. As eosinophilic infiltration of the intestinal mucosa is not specific to EE, we must consider other differential diagnoses in patients with gastrointestinal symptoms and eosinophilia. In our case, we have ruled out the possibility of intestinal parasites by direct stool examination and inflammatory bowel disease by performing an endoscopy. It is also necessary to exclude a hypereosinophilic syndrome, which is defined by peripheral blood eosinophilia of >1500 eosinophils/mm for more than 6 months that is associated with the involvement of at least 1 organ such as the heart, lungs, skin, or bone marrow.^[16]^ In our patient, the auxiliary examination results were unremarkable. Thus, we considered the possibility of hypereosinophilic syndrome to be less likely. We will do more further research on such diseases.

The symptoms of EE are dependent on the extent of eosinophilic infiltration within the affected bowel wall.^[3]^ Eosinophils may infiltrate the mucosal layer, the muscular layer, or the subserosal layer of the GI tract.^[2]^ Initially, EE was classified into 3 different forms according to the predominantly involved intestinal layer: the mucosal form, which is characterized by a predominantly mucosal inflammation accompanied by diarrhea, abdominal pain, and signs of malabsorption or protein-losing enteropathy; the muscular form, which is characterized by intestinal strictures with symptoms such as abdominal pain, nausea, and vomiting that can lead to intestinal occlusion; and the subserosal form, which is characterized by eosinophilic-rich ascites accompanied by bloating and abdominal pain.^[7]^ One study has reported that 44%, 12%, and 49%, of patients with EE were of the mucosal form, muscular form, and subserosal form, respectively.^[16]^ In this patient, abdominal pain was the main clinical manifestation; however, the pathological examination did not show the extent of eosinophilic infiltration within the affected bowel wall. This aspect in the evaluation of the disease needs to be improved upon.

The pathophysiology of EE is still unclear. However, it has been reported that it is an immune-mediated food antigen-driven disorder characterized by delayed IgE-mediated Th2-type immune responses.^[17]^ Patients diagnosed with EE often have a history of allergic disorders including asthma, eczema, seasonal allergies, and food allergies. This suggests hypersensitivity as the etiology of the disease,^[11]^ which may activate and promote the differentiation of interleukin (IL)-5 via Th2 cells, resulting in eosinophilic infiltration of the gut.^[18]^ However, food intolerance or a history of allergies is not usually present and IgE levels are less frequent. In our case, the patient did not have any type of allergy. It has been reported that eosinophils play a critical role in the pathogenesis of EE.^[17]^ Activated eosinophils can secrete a large number of pro-inflammatory cytokines such as IL-4, IL-5, IL-13, and chemokine regulated upon activation normal T-cell expressed and secreted,^[19]^ which could recruit and activate other adaptive immune cells to the site of inflammation. In this case, the elevated ratio of eosinophils was seen in the peripheral blood, lamina propria of the intestinal mucosa, and bone marrow, which was inconsistent with published studies.

Treatment strategies of EE focus on either medical or dietary therapy, with the aim of not only controlling symptoms and inflammation but also identifying potential food triggers. Due to the rarity of the disease, there are no established therapeutic strategies for EE. Hence, treating EE is challenging. However, approximately 40% of patients will have spontaneous remission.^[20]^ In clinical practice, the therapeutic methods mainly include dietary therapy, corticosteroids, immunosuppressive drugs, and biological therapies. Steroids, which are generally used for a short period to reduce inflammation, have been the mainstay of medical treatment and has exhibited good response rates.^[21]^ Although corticosteroid treatment alone has been reported to result in clinical remission in 50% to 90% of patients with EE,^[12,22]^ about 20% will still require low-dose prednisone to maintain clinical remission due to corticosteroid dependency. Since high doses of steroids are associated with systemic side effects, the goal of steroid therapy in EE is to decrease the severity of symptoms using the least possible dose rather than using high doses to control tissue eosinophilia. In our case, the patient had good response to steroid treatment, resulting in a good prognosis. Patients who fail to respond to steroids should undergo careful reevaluation to rule out the presence of an underlying infection or alternate diagnoses (e.g., inflammatory bowel diseases). Several other approaches for the treatment of recurrent or refractory symptoms have been described in case reports or small series. Azathioprine has been used with efficacy in patients with EE who are either dependent on, or refractory to, glucocorticoids.^[7]^ In addition, leukotriene inhibitors, mast cells stabilizers, antihistamines, and sodium cromoglycate have also been used to treat patients with EE.^[23–28]^ Biological therapies targeting the eosinophilic signaling pathway such as mepolizumab, an anti-IL-5 antibody, or omalizumab, an anti-IgE monoclonal antibody, have also been reported to be potential therapeutic agents for EE.^[7]^ However, because of the limited data available, none of these agents can be recommended for routine use.

## 4 Conclusion

EE is a rare and heterogeneous disease that requires a high degree of suspicion and an endoscopic biopsy for definite diagnosis; hence, the disease may probably be underdiagnosed in clinical practice. From this case, we have learned a number of things: The dynamic changes of the eosinophil count should be monitored because it may be normal at the beginning of the disease; EE should be distinguished from tumors, hematologic diseases, and most especially, eosinophilia; Not all patients with EE have a history of allergies; multisite biopsies should be carried out if EE is suspected to lessen the frequency of follow-up examinations of patients, reduce medical costs, and improve their quality of life. Although our patient had good response to steroid treatment, not all patients with EE will have a good response to steroids.

## Acknowledgments

The authors would like to give their heartfelt thanks to the patient, Sun Song, for his contribution in providing very important clinical data to this article.

## Author contributions

**Data curation:** Cuimei Ma, Zhen Xu.

**Formal analysis:** Yibo Wang.

**Methodology:** Yuan Xue.

**Resources:** Zongjing Hu.

**Writing – review & editing:** Guangxi Zhou, Hairong Zhang.
